# Incidence, Timing, and Pattern of Atypical Recurrence after Minimally Invasive Surgery for Urothelial Carcinoma

**DOI:** 10.3390/jcm13123537

**Published:** 2024-06-17

**Authors:** Gabriele Bignante, Celeste Manfredi, Francesco Lasorsa, Angelo Orsini, Leslie Claire Licari, Eugenio Bologna, Daniel F. Roadman, Daniele Amparore, Giuseppe Lucarelli, Luigi Schips, Cristian Fiori, Francesco Porpiglia, Riccardo Autorino

**Affiliations:** 1 Department of Urology, Rush University Medical Center, Chicago, IL 60612, USA; gabrielebignante@gmail.com (G.B.); francesco-lasorsa96@libero.it (F.L.); angelo_orsini@rush.edu (A.O.); leslieclaire.licari@uniroma1.it (L.C.L.); eugenio.bologna@uniroma1.it (E.B.); daniel_roadman@rush.edu (D.F.R.); ricautor@gmail.com (R.A.); 2 Division of Urology, Department of Oncology, University of Turin, San Luigi Gonzaga Hospital, 10043 Turin, Italy; danieleamparore@hotmail.it (D.A.); cristian.fiori@unito.it (C.F.); francesco.porpiglia@unito.it (F.P.); 3 Unit of Urology, Department of Woman, Child and General and Specialized Surgery, University of Campania “Luigi Vanvitelli”, 80131 Naples, Italy; 4 Andrology and Kidney Transplantation Unit, Department of Precision and Regenerative Medicine and Ionian Area-Urology, University of Bari “Aldo Moro”, 70124 Bari, Italy; giuseppe.lucarelli@uniba.it; 5 Urology Unit, Department of Medical, Oral and Biotechnological Sciences, “G. d’Annunzio” University, 66100 Chieti, Italy; luigischips@hotmail.com; 6 Department of Maternal-Child and Urological Sciences, Sapienza University Rome, Policlinico Umberto I Hospital, 00161 Rome, Italy

**Keywords:** atypical recurrences, metastasis, minimally invasive, nephroureterectomy, radical cystectomy, urothelial carcinoma

## Abstract

The management of urothelial carcinoma has evolved with the introduction of minimally invasive techniques such as laparoscopic or robotic procedures, challenging the traditional approach of open surgery, and giving rise to atypical recurrences (ARs). ARs include port-site metastasis and peritoneal carcinomatosis, yet discrepancies persist among authors regarding their precise classification. Incidence rates of ARs vary widely across studies, ranging from less than 1% to over 10% in both muscle-invasive bladder cancer (MIBC) and upper tract urothelial tumor (UTUC). Peritoneal metastases predominate as the most common ARs in patients with MIBC, while retroperitoneal metastases are prevalent in those with UTUC due to differing surgical approaches. The timing of AR presentation and survival outcomes closely mirror those of conventional recurrences, with which they are frequently associated. Pneumoperitoneum has progressively been regarded less as the cause of ARs, while surgical-related risk factors have gained prominence. Current major surgical-related causes include tumor spillage and urinary tract violation during surgery, avoidance of endo bag use for specimen extraction, and low surgical experience. Factors such as tumor stage, histological variants, and lympho-vascular invasion correlate with the risk of ARs, suggesting a close association with tumor biology. Further studies are required to better understand the incidence, risk factors, characteristics, and outcomes of ARs.

## 1. Introduction

Urothelial carcinoma has traditionally been managed using open surgery techniques, considered the safest and most established treatment approach. However, the emergence of minimally invasive techniques, such as laparoscopic or robotic procedures, has introduced new surgical modalities for treating this carcinoma [1,2]. Nevertheless, the adoption of these techniques has sparked significant debate.

A recent Cochrane systematic review [3] recognizes robot-assisted radical cystectomy (RARC) as a secure and feasible approach for treating nonmetastatic muscle-invasive bladder cancer (MIBC). This approach provides similar 90-day complication rates, surgical margin rates, median-term oncological outcomes, and quality-of-life outcomes [1]. Nevertheless, the use of laparoscopic or robot-assisted radical nephroureterectomy (LRNU or RARNU) for upper urinary tract urothelial carcinoma (UTUC) is still under debate [4,5,6]. In the latest update of the European Association of Urology (EAU) guidelines, open radical nephroureterectomy (ORNU) with bladder cuff excision remains the standard treatment for invasive or large (cT3/cT4/N+/M+) tumors, while minimally invasive procedures may be considered for less invasive cases [2].

However, with the introduction of laparoscopic and robotic surgery, certain types of spread not previously identified with open surgery have been noted. Specifically, peritoneal carcinomatosis and dissemination along the trocar pathway have been noted since 1978 [7], leading to the definition of atypical recurrence (AR). The term “AR” highlights the contrast with typical recurrences, including both local and distant metastases. Currently, data on the topic are limited, therefore, any effort to increase available evidence could improve the management and outcomes of patients with urothelial cancer.

The primary aim of this study is to provide a comprehensive narrative review of the current evidence on ARs after minimally invasive surgery for urothelial carcinoma, focusing on their incidence, timing, and pattern. In addition, this article aims to discuss the possible surgical and tumor factors related to the occurrence of ARs.

## 2. Materials and Methods

### 2.1. Literature Search Strategy

We conducted a comprehensive bibliographic search on PubMed, Scopus, and EMBASE databases in April 2024 without chronological restrictions.

The following keywords were variously combined using Boolean operators to identify relevant articles by title and abstract: atypical, abnormal, anomalous, unconventional, exceptional, port, port-site, peritoneal, peritoneum, trocar, urothelial, ureter, calyces, pelvis, UTUC, bladder, nephroureterectomy, cystectomy, tumor, cancer, carcinoma, neoplasm, laparoscopic, robot, robotic, robot-assisted, mini-invasive, minimally invasive.

In addition, the reference lists of the articles found were screened manually to include other relevant articles.

Finally, we included 10 articles on cystectomy and 8 papers on nephroureterectomy in the review.

### 2.2. Inclusion and Exclusion Criteria

The following eligibility criteria were established: (1) articles published in English, (2) including patients with urothelial carcinoma of the bladder or upper urinary tract undergoing laparoscopic- or robot-assisted cystectomy or nephroureterectomy (compared or not with open surgery), (3) reporting data on ARs (incidence, timing, or pattern).

Studies were excluded based on the following criteria: (1) pure non-urothelial histology, (2) preoperative metastatic disease, (3) cystectomy for non-MIBC, (4) malignant tumors in other sites, (5) conference abstracts, letters, comments, replies, narrative reviews.

### 2.3. Data Extraction and Synthesis

From each eligible original article, we extracted the following details: name of the first author, publication year, study type, study period, number of patients, diagnosis, surgical procedure, length of follow-up, data on ARs (incidence, site, timing), and survival. In addition, evidence of possible surgical and tumor factors associated with ARs were reported. Relevant data deriving from systematic reviews and meta-analyses were also discussed.

The results of the selected studies were presented in a narrative manner without manipulation or quantitative synthesis (meta-analysis) of the data. However, we summarized some results using sums and percentages to get a better overview of the evidence.

## 3. Evidence Synthesis

### 3.1. Definition of AR

The EAU MIBC guidelines define AR as “one or a combination of the following presentations: port-site metastasis or peritoneal carcinomatosis” [1], whereas no specific definition exists in the UTUC guideline. In general, there is a lack of consensus among authors regarding the precise definition of AR: some adhere strictly to the guidelines, considering only peritoneal carcinomatosis and port-site metastases as ARs, others extend this definition to include additional types of secondary occurrences.

For instance, Kubota et al. [8] classified two soft tissue metastases following laparoscopic or robotic cystectomy as ARs. Specifically, Kubota reported a gluteal muscle metastasis and an inguinal region metastasis in a patient who also presented with peritoneal carcinomatosis. Additionally, Tan et al. [9] reported an exceedingly rare metastasis to the penis following RARC.

In case of LRNU, Kanno et al. [10] observed retroperitoneal carcinomatosis in 15 cases. This observation was further supported by Franco et al. [11] who analyzed the ROBUUST (ROBotic surgery for Upper tract Urothelial cancer STudy) data, comprising a large series of LRNU and RARNU. Carrion et al. [12] identified two subcutaneous lesions separate from the incision scars and two abdominal wall lesions as ARs following LRNU.

Considering these different observations, it is clear that there is significant complexity in establishing a universally accepted definition of AR. Consequently, it remains difficult to correctly interpret and accurately compare studies on ARs. Standardization would be desirable to improve knowledge on the topic. In our opinion, they could be defined as recurrences after surgical treatment for curative purposes in locations other than the primary localization and the most frequent localizations of distant metastases. Furthermore, they could be classified into peritoneal, retroperitoneal, related to port sites, related to stoma site, abdominal wall, and involving other organs.

### 3.2. Incidence, Pattern, and Timing of ARs

ARs are infrequent after minimally invasive surgery for MIBC, with reported incidence rates ranging from less than 1% to more than 10% across different studies. Collins et al. and Hussein et al. [13,14] observed an AR rate of 1–2% after RARC in retrospective studies involving 717 and 1380 patients, identifying only peritoneal and port-site metastases. However, a higher rate of AR was found by Kubota et al. [8] in a cohort of 63 patients. Interestingly, higher AR rates were found in smaller patient series [8,15].

A similar incidence rate was found in patients with UTUC. Indeed, Ariane et al. and Kang et al. [16,17] observed AR in 2% of patients in large series. On the contrary, Carrion et al. [12] reported a higher incidence of ARs (7%) in a series of 117 LRNU cases. Similarly to patients with MIBC, the incidence of ARs among those treated for UTUC ranges from 1 to 7%.

In patients with MIBC, peritoneal metastases were identified in 84% of cases with ARs and were reported by almost all authors. Furthermore, port-site metastases were the second most common AR, occurring in 14% of patients. Conversely, soft tissue and stoma site metastases were rare occurrences.

When considering patients with UTUC, a different distribution of ARs was observed. More than half of UTUC patients with ARs presented with retroperitoneal metastases (53%), while 24% exhibited peritoneal recurrences. Additionally, the rate of port-site metastases was higher in UTUC patients compared to those with MIBC, with only a few cases of port-site and subcutaneous secondaries.

**Figure 1:**
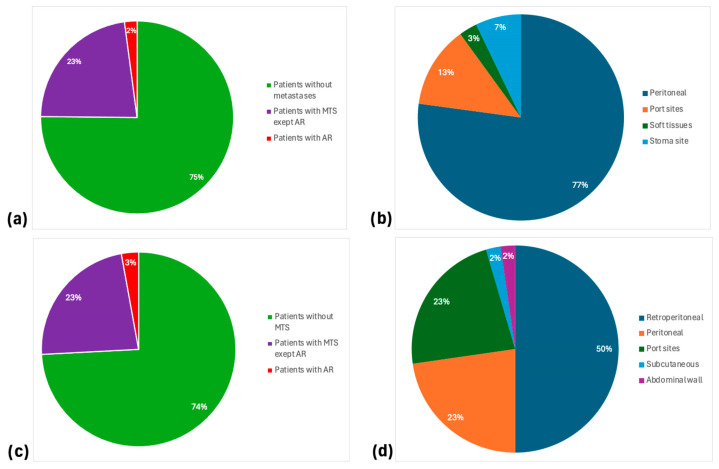
Proportions and sites of ARs after minimally invasive surgery for MIBC and UTUC. (**a**) Proportion of MTS and ARs among patients with MIBC; (**b**) Distribution of ARs among patients with MIBC (**c**) Proportion of MTS and ARs among patients with UTUC; (**d**) Distribution of ARs among patients with UTUC. AR: Atypical Recurrence; MIBC: Muscle-Invasive Bladder Cancer; MTS: metastasis; UTUC: Upper Urinary Tract Urothelial Carcinoma.

These data indicate that the peritoneum or retroperitoneum serves as the primary site of AR in both groups of patients, depending on the transperitoneal or retroperitoneal approach. Finally, when considering the incidence of peritoneal and retroperitoneal involvement among all patients, it ranges from 1% to 10% in MIBC cases and up to 6% in patients with UTUC. This distribution is illustrated in Figure 1.

To better understand the incidence rate of ARs, it should not be separated from the analysis of the total recurrences. In several articles, it was noted that ARs were not the only recurrence presentation. Indeed, in most cases, ARs were associated with distant metastases [17,18]. However, determining the true incidence of ARs is a challenging task for different reasons. Jancke et al. [19] suggested that ARs may be more prevalent than reported in the literature due to publication bias. Even among the studies included in this review, there is a notable heterogeneity in data presentation: while some studies encompass all ARs, others focus exclusively on one type of AR.

The available data on the timing of AR presentations is very limited. Only a few studies have analyzed the duration between minimally invasive surgery for urothelial carcinoma and AR onset. Regarding patients who underwent RARC, the median time, observed in only 4 studies [8,9,14,20], varied from 3 to 11 months, with Nguyen et al. [20] reporting a maximum of 17 months after surgery. In the same study, Nguyen observed that the time to recurrence did not significantly differ in patients with ARs compared to those with local or distant recurrence. Timing after surgery to recurrence has also been observed in 4 studies on UTUC [17,21,22,23], and data do not differ widely from patients with MIBC. In these cases, a median range of 3.5–7 months after surgery is needed for AR onset.

Similarly, there is a paucity of data on survival times after ARs. Among studies on MIBC, only Kubota et al. [8] reported survival from surgery to death, with a median of 9.3 months and a range from 3 to 25 months. Similar survival times were observed in patients with UTUC, with median survival from surgery ranging from 2.9 to 9.9 months [10,12], and a maximum reported survival of 33 months was described by Carrion et al. [12]. Conversely, only Kang et al. [17] observed the time from ARs presentation to death in 4 patients. These patients survived a median of 7 months from the onset of the port-site metastases.

Given that patients with ARs often have concurrent local or distant metastases, it becomes challenging to ascertain whether the reduced survival is directly attributable to ARs or not. However, it is well-established that AR tends to be associated with a rapidly progressive form of the tumor [24]. Consequently, the interval between surgery and AR or AR and death is reasonably short.

The main features of the studies included in the review and the characteristics of the ARs identified by them are summarized in Table 1 and Table 2.

### 3.3. Surgery-Related Risk Factors

Factors related to the surgical technique might also have an impact on the onset of ARs.

Pneumoperitoneum was initially blamed as the primary cause of cell spreading during minimally invasive surgery for urothelial carcinoma. Kazemier et al. [25] reported the “chimney effect”: carbon dioxide leaks alongside trocars result in significant local gas flow at trocar wounds. This flow may carry aerosolized tumor cells, therefore, continuous air leakage around the port could increase the presence of tumor cells at the port site, facilitating metastases in this location. Other evidence from animal studies indicated that the establishment of pneumoperitoneum may potentially heighten the likelihood of cancer cell dissemination within the peritoneal cavity by inhibiting the peritoneal immune response against malignant urothelial cells [26]. However, further analyses have shown that ARs seem to be primarily associated with adherence to principles of safe oncological surgery and tumor-related causes [20].

Avoiding tumor spillage is crucial for ensuring oncological safety during surgery. Hussein et al. [14] conducted a survey among leading surgeons and found that ARs were associated with urine spillage during surgery. They noted that urine spillage during RARC could occur in cases of extravesical disease, extensive nodal involvement, or due to technical error. Regardless of the cause, urine spillage could potentially lead to seeding of the peritoneal cavity. Similarly, Manabe et al. [21] observed a case of port-site recurrence during LRNU due to urine extravasation resulting from urinary tract obstruction by ureteral cancer. Despite intraoperative adverse events being widely recognized as prognostic factors for recurrence, Carrion et al. [12] observed that these events are often inadequately reported and rarely scrutinized in most studies.

Another surgical aspect to be considered during laparoscopic and robot-assisted procedures is the use of an endo bag to collect the removed organ. The efficacy of using bags to extract specimens from the operative site is a well-established step in surgery, widely practiced across various specialties [27,28]. Ariane et al. [16] observed three port-site metastases over 150 cases of LRNU, all before the adoption of laparoscopic bags for specimen extraction. The use of endo bags is now recognized as a fundamental aspect of surgical practice to ensure oncological safety and reduce the risk of recurrence. Nearly all the studies included in this review acknowledge their use.

**Table 1:** Atypical recurrences in patients with muscle-invasive bladder cancer.

Author	Type of Study	Study Period	Patients (n)	Procedure	Follow-Up Period (Median (Min–Max or IQR) Months)	Total Recurrences (n (%))	Atypical Recurrences (n (%))	Atypical Recurrences Sites (n (%))	Time from Surgery to AR (Median (Min–Max or IQR) Months)	Survival from Surgery (Median (Min–Max or IQR) Months)
Simone [6]	Prospective	2003–2006	40	LRC	60	5 (12)	1 (2)	Port sites: 1 (2)	N/A	N/A
Collins [13]	Retrospective	2003–2015	717	RARC	24	182 (25)	7 (1)	Peritoneal: 5 (1) Port sites: 2 (0)	N/A	N/A
Gandaglia [29]	Retrospective	2004–2014	155	RARC	42 (33.2–50.7)	83 (54)	3 (2)	Peritoneal: 3 (2)	N/A	N/A
Nguyen [20]	Retrospective	2001–2015	310	RARC	24 (14–51) ^b^	81 (26)	13 (4)	Peritoneal: 13 (4)	11 (3–17) ^b^	N/A
Tan [9]	Retrospective	2011–2014	90	RARC	16.1 (11.2–27.0) ^b^	17 (19)	3 (3)	Peritoneal: 2 (2) Port sites: 1 (1)	8.5 (4.1–16.1) ^#,b^	N/A
Hussein [14]	Retrospective	2003–2016	1380	RARC	24	305 (22)	22 (2)	Peritoneal: 17 (1) Port sites: 5 (1)	3	N/A
Bochner [15]	Prospective	2010–2013	60	RARC	59 (47–71) ^b^	20 (33)	5 (8)	Peritoneal: 5 (8) Stoma site: 5 (8)	N/A	N/A
Niegisch [18]	Retrospective	2008–2016	89	RARC	32 (23–39) ^b^	10 (11)	1 (1)	Peritoneal: 1 (1)	N/A	N/A
Venkatramani [24]	Prospective	N/A	150	RARC	36	39 (26)	2 (1)	Peritoneal: 2 (1)	N/A	N/A
Kubota [8]	Retrospective	2007–2018	63	LRC/RARC	29	17 (27)	7 (11)	Peritoneal: 6 (10) Port sites: 1 (2) Soft tissues: 2 (3)	5.5 (2.6–11.8) ^a^	9.3 (3.3–25.2) ^a^

LRC: Laparoscopic Radical Cystectomy; IQR: Interquartile Range; N/A: Not available; RARC: Robot-Assisted Radical Cystectomy. ^a^: median (min–max); ^b^: median (IQR); ^#^: values referred to the total number of patients (not just the atypical ones).

**Table 2:** Atypical recurrences in patients with upper tract urothelial carcinoma.

Author (Year)	Type of Study	Study Period	Patients (n)	Procedure	Follow-Up Period (Median (Min–Max or IQR) Months)	Total Recurrences (n (%))	Atypical Recurrences (n (%))	Atypical Recurrences Sites (Number (%))	Time from Surgery to AR (Median (Min–Max or IQR) Months)	Survival from Surgery (Median (Min–Max or IQR) Months)
Manabe [21]	Retrospective	2000–2004	58	LRNU	N/A	30 (52)	1 (2)	Port sites: 1 (2)	6,4	N/A
Ariane [16]	Retrospective	1995–2010	150	LRNU	27 (10–48) ^#,b^	21 (14)	3 (2)	Port sites: 3 (2)	N/A	N/A
Carrion [12]	Retrospective	2007–2012	117	LRNU	20 (3–97) ^a^	36 (31)	8 (7)	Peritoneal: 5 (4) Subcutaneous: 2 (2) Abdominal wall: 2 (2) Port sites: 2 (2)	N/A	2.9 (1.5–33.4) ^a^
Kang [17]	Retrospective	2013–2018	240	LRNU	12.6 (3–45) ^a^	N/A	4 (2)	Port sites: 4 (2)	4.3 (1–8) ^a^	7 (2–17) ^a,c^
De Groote [22]	Retrospective	2008–2017	78	RARNU	15	22 (28)	1 (1)	Peritoneal: 1 (1)	7 (4–7) ^b^	N/A
Morselli [23]	Retrospective	2008–2019	47	LRNU	89.3	18 (38)	3 (6)	Peritoneal: 3 (6)	3.5 (3–4)	N/A
Kanno [10]	Retrospective	2002–2020	283	LRNU	31	N/A	14 (5)	Retroperitoneal: 12 (4) Port sites: 5 (2)	N/A	9.9
Franco [11]	Retrospective	2015–2023	1935	LRNU (779) RARNU (1156)	28 (14–48) ^#,b^	624 (32)	49 (3)	Retroperitoneal: 32 (2) Port sites: 6 (0) Peritoneal: 11 (1)	N/A	N/A

LRNU: Laparoscopic Radical Nephroureterectomy; IQR: Interquartile Range; N/A: Not available; RARNU: Robot-assisted Radical Nephroureterectomy. ^a^: median (min–max); ^b^: median (IQR); ^c^: survival from AR onset; ^#^: values referred to the total number of patients (not just the atypical ones).

The association between the surgical experience of the center and individual operators and the oncological outcomes is well-established in the literature [30]. Hussein et al. [14] observed a decline in oncological recurrence rate after RARC from 10% in 2006 to 6% in 2015. Jancke et al. [19] noted that five out of eight cases of ARs following RARC occurred within the initial 100 patients treated robotically. Complex surgeries, particularly those performed with minimally invasive techniques, require a lengthy learning curve during which surgical errors may occur. Common findings during initial cases include violations of the urinary tract, resulting in urine spillage, tumor edge infractions, and small negative margins [31]. Consequently, EAU recommends centralizing patients undergoing cystectomy in high-volume centers [2]. These observations may suggest a theoretically higher incidence of ARs in low-volume centers, which, however, are probably largely underreported.

In summary, some reasonable principles of safe oncological surgery to be adopted during minimally invasive surgery for urothelial tumors to reduce the risk of ARs could be as follows: (1) avoiding entering the urinary tract to prevent urine spillage during the procedure, (2) minimizing direct contact between surgical instruments and the tumor (3) avoiding morcellation of the tumor, (4) utilizing an endo bag for tumor extraction, (5) performing the procedure in a closed system.

### 3.4. Tumor-Related Risk Factors

Several characteristics of urothelial tumor appear to correlate with the risk of ARs. Nguyen et al. [20] were among the first to demonstrate that tumor stage in MIBC was the most potent predictor of recurrence, whether local, distant, or atypical in location. Similarly, Carrion et al. [12] found that among eight patients who underwent LRNU and developed ARs, five were at advanced tumor stages (pT3/pT4), while the other two had pT2 tumors associated with urinary tract violation during surgery. In seven of these cases, ARs were linked to concurrent local or distant typical recurrences. Kang et al. [17], who observed four port-site metastases out of 240 LRNU cases, noted that three of these patients had pT3 tumors, suggesting that tumor aggressiveness, as indicated by grade and stage, could contribute to tumor seeding propensity. Additionally, all four patients presented with multiple distant recurrences simultaneously with ARs. All these findings suggest that ARs from tumor seeding may be closely associated with tumor biology.

Histological variants of urothelial carcinoma are generally considered to be indicative of a poor prognosis. Kubota et al. [8] observed the presence of histological variants or differentiation other than pure urothelial carcinoma in three out of seven patients with ARs after minimally invasive cystectomy. Specifically, they observed squamous differentiation, neuroendocrine differentiation, and plasmacytoid variants. Among all the patients who underwent transurethral resection of the bladder (TURB) at that center, only these three patients had histological variants reported in their pathology results. Subsequently, after radical cystectomy, all of them exhibited ARs. These findings support the idea that aggressive histological variants may be associated with the onset of ARs.

Another pathological finding related to the development of ARs is lympho-vascular invasion (LVI). Janke et al. [19] observed eight cases of port-site metastases after RARC. In their report, LVI was present in seven patients, while the eighth exhibited perineural growth. Furthermore, the author noted that all cases involved patients with distant metastases associated with ARs. The role of LVI as a prognostic factor for progression is still controversial [32]. A meta-analysis observed a strong association between LVI and disease recurrence in pN0 patients and a poor prognosis in cases of advanced tumor stage (pT3/pT4) [33]. However, while this study considered all recurrences, only Nguyen et al. [20] confirmed the correlation between LVI and ARs in patients with MIBC, noting that LVI was one of the strongest independent predictors. Nonetheless, given that most patients with ARs have synchronous distant metastases, it would be challenging to determine whether LVI is associated with ARs or only distant recurrences. Key points of the article are summarized in Table 3 and Table 4.

### 3.5. Limitations

The results of our review should be read taking into account several limitations of the current literature.

First, despite the well-defined search and article selection criteria, this is not a systematic review and therefore some relevant papers may not have been included. Consequently, quantitative synthesis of data was not carried out; however, the high heterogeneity and low quality of the studies included would have made the results unreliable.

Furthermore, the absence of a univocal definition of AR may have led to misclassification of some recurrences or failure to include some studies.

Finally, considering that most of the articles included in this study are retrospective, a cause–effect relationship could not be assessed.

## 4. Conclusions

The occurrence of ARs after minimally invasive surgery for urothelial cancer is rare, with an incidence generally lower than 10%, which tends to increase in low-volume centers. ARs commonly manifest as peritoneal or retroperitoneal metastases, depending on the surgical approach, followed by port-site metastases. These occurrences are typically associated with more aggressive tumors, histological variants, or local and distant metastases. The timing of AR presentation and survival outcomes are similar to those of local and distant recurrences. Adherence to strict oncological safety protocols during surgical procedures, such as preventing urine spillage, avoiding direct instrument–tumor contact, and utilizing endo bags for specimen extraction may help mitigate the risk of AR occurrence. Further studies are needed to better elucidate the incidences, risk factors, characteristics, and outcomes of ARs.

## Figures and Tables

**Table 3:** Key points from MIBC and UTUC patients.

	MIBC	UTUC
Oncological safety of laparoscopic and robot-assisted procedures	Yes	Yes (cT1/cT2,cN0,cM0) Unclear (cT3/cT4/N+/M+)
Incidence of ARs (% range)	1–11%	1–7%
Pattern (% of all ARs)	Peritoneal: 77% Port sites: 13% Stoma site: 7% Soft tissues: 3%	Retroperitoneal: 50% Peritoneal: 23% Port sites: 23% Subcutaneous: 2% Abdominal wall: 2%
Timing from surgery to AR onset (median range)	5.5–11 months	3.5–7 months
Survival from diagnosis to death (median range)	9.3 months	2.9–7 months

**Table 4:** Key points on surgical- and tumor-related risk factors.

Surgery-Related Risk Factors	Tumor-Related Risk Factors
Tumor spillage Urinary tract violation Omitting the use of an endo bag Low surgical experience Low-volume center	Advanced tumor stages (pT3/pT4) Distant metastases Histological variants Lymphovascular invasion (LVI)

## Data Availability

This is a review, therefore the data reported can be found in the cited articles.
